# Supplementary material for: Artificial photosynthetic cell producing energy for protein synthesis
Source: Nat Commun. 2019 Mar 22;10:1325. doi: 10.1038/s41467-019-09147-4 (PMC6430821; doi:10.1038/s41467-019-09147-4)
Supplement: Supplementary file 1 — Supplementary Information [file 41467_2019_9147_MOESM1_ESM.pdf]

Supplementary Information for Artificial photosynthetic cell producing energy for protein synthesis

Samuel Berhanu, Takuya Ueda, Yutetsu Kuruma

Email: kuruma@elsi.jp

Supplementary Figure 1 to 24
Supplementary Table 1 to 5
References

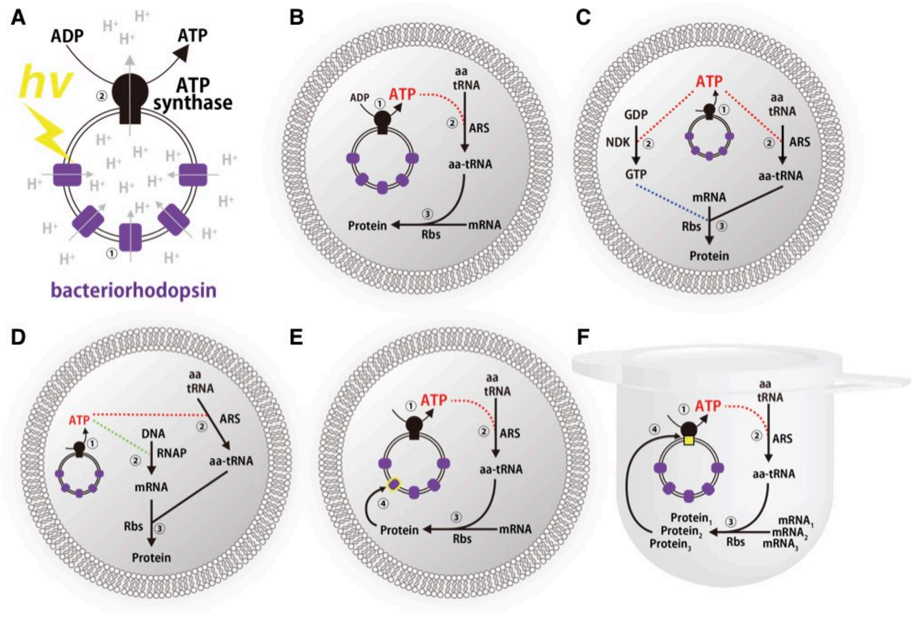

Supplementary Figure 1. Schematics of photosynthetic reaction in GUV or in vitro. (A) Light-driven ATP synthesis by artificial organelle consists of bacteriorhodopsin (bR) and ATP synthase (F_oF_1). **(B)** Photosynthesized ATP were consumed as energy for aminoacylation of tRNA by aminoacyl-tRNA synthetase (ARS). The resulting amino acid (aa)-tRNA is subsequently consumed for translation by ribosome (Rbs). **(C)** Photosynthesized ATP were consumed as energy for aminoacylation of tRNA and for phosphorylation of guanosine diphosphate (GDP), resulted in guanosine triphosphate (GTP), by nucleoside-diphosphate kinase (NDK). The resulting GTP were consumed as energy for translation. **(D)** Photosynthesized ATP were consumed as energy for aminoacylation of tRNA and as substrate of transcription by RNA polymerase (RNAP), then, follows as same as (C). **(E)** Photosynthesized ATP were consumed as same as (B) to synthesize *de novo* bR that becomes a part of the artificial organelle. **(F)** Photosynthesized ATP were consumed as same as (B) to synthesize three component proteins of F_o complex (the membrane embedding part of F_o F_1). The resulting de novo F_o become a part of the artificial organelle. (B)–(E) were performed inside giant vesicle and (F) was performed in vitro. The numbers described in figures indicate the order of reactions.

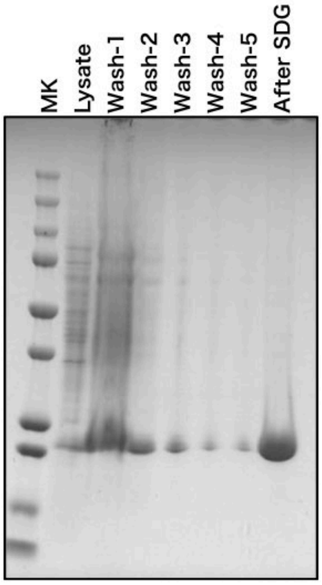

Supplementary Figure 2. SDS-PAGE analysis of purple membrane. Purple membrane prepared from *Harboracterium salinarum* was washed 5 times with buffer and subjected by ultracentrifugation with sucrose density gradient (SDG). All samples were analyzed by 15 % SDS-PAGE. The sizes of marker proteins (MK) indicate 250, 150, 100, 75, 50, 37, 25, 20, 15, 10 KDa from the top. The theoretical size of bR is 26.8 kDa. The gel was stained by Coomassie Brilliant Blue.

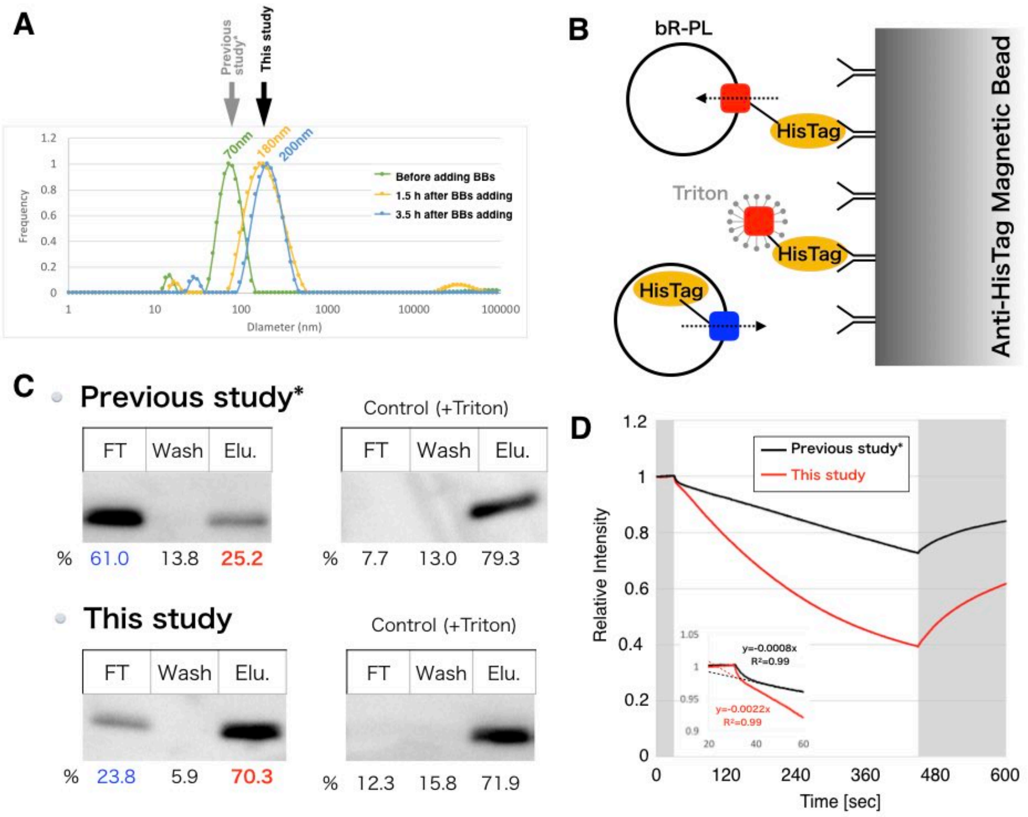

Supplementary Figure 3. Optimized bR-PLs preparation method. (A) The timing of bR addition. bR-PLs were prepared by adding the purified bR into lipid-detergent mixture, then followed by removing detergent with BioBeads (BBs). In the previous study (*[1]), bR was mixed before adding BBs, where the value of dynamic light scattering (DLS) measurement indicating 70nm (dia.). On the other hand, in this study, bR was mixed at 1.5 hour after the BBs addition, where DLS value indicating 180nm. **(B)** bR-PLs were prepared with 0.2 µM recombinant bR containing HisTag at the C-terminus and 41.3 mM lipid mixture (SoyPC extract/ cholesterol within the molar ratio 70/30). After mixing with NiNTA-conjugating magnetic beads in the presence or absence of TritonX-100, the flow through (FT), wash, and elution (Elu.) fractions were collected and analyzed by SDS-PAGE. **(C)** The resulting FT, wash, and Elu. fractions were analyzed by SDS-PAGE and western-blotting using anti-HisTag antibody. The bR appeared in the Elu. fraction represents that C-terminus of bR is facing to the outward of PLs, whereas the bR in the FT fraction represents that bR is facing to the inward. Band intensities were measured by ImageGauge software (Fujifilm) and shown at the bottom of the gel. Triton-X100 was used to solubilize PLs for control experiment. **(D)**

The light-driven proton-pump activity of both bR-PLs were compared. The inset indicates initial rate of the each PLs.

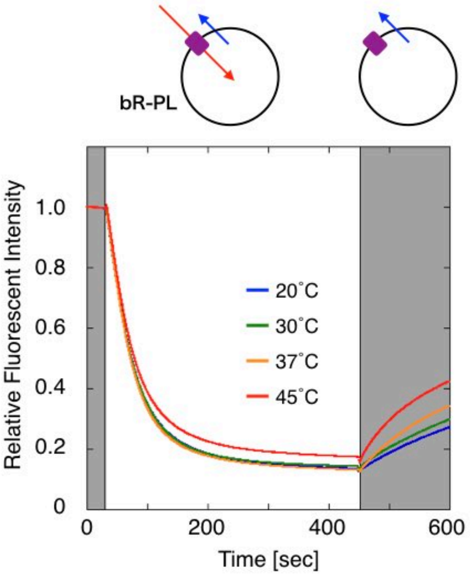

	H+-leak		H+-pump	
Temp (˚C)	Slope (R.F.I./s)	R^2	Slope (R.F.I./s)	R^2
45	0.0031	0.997	−0.0131	0.999
37	0.0022	0.997	−0.0140	0.999
30	0.0016	0.997	−0.0138	0.999
20	0.0013	0.999	−0.0135	0.999

Supplementary Figure 4. Proton leak from inside to outside of bR-PLs. bR-PLs were prepared from 20 µM bR and 16 mg/ml soybean extract. Light-dependent proton-pump assay was performed at different temperatures. The gray and white areas indicate dark and light condition, respectively. The proton concentration at the outside of PLs was monitored using proton indicator ACMA (ex 410nm/em 480nm). At the 450 sec, light was turned off and the leak of the pumped proton were measured. The initial slopes (Relative Fluorescent Intensity/sec) of H^+-leak and H^+-pump are obtained from the periods of 454-474 sec and 35-55 sec, respectively.

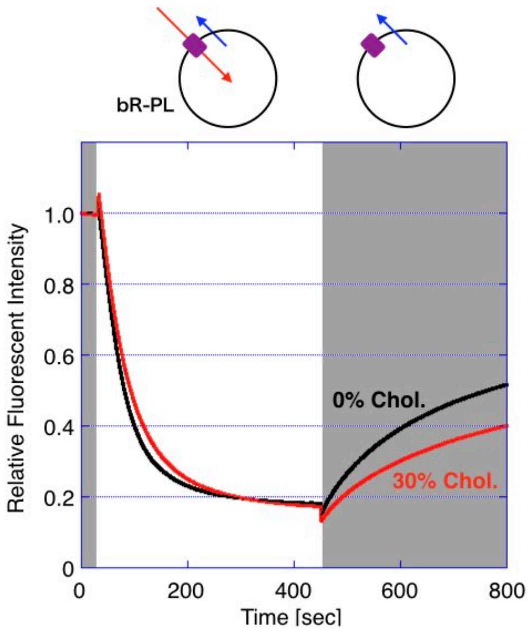

Chol.	Slope (in Dark)	R^2
0%	0.0028	0.99
30%	0.0019	0.99

Supplementary Figure 5. Effect of cholesterol against proton leak. Proton-pump activity of bR-PLs and its leak in the dark condition (gray area) was measured in the presence or absence of 30 % cholesterol (chol.) in the lipid composition of bR-PLs. bR-PLs were prepared as described in Supplementary Figure 3. The proton concentration at the outside of PLs was monitored using proton indicator ACMA (ex 410nm/em 480nm). At the 450 sec, light was turned off and the leak of the pumped proton was measured. The initial slopes of proton leak are obtained from the periods of 451-470 sec.

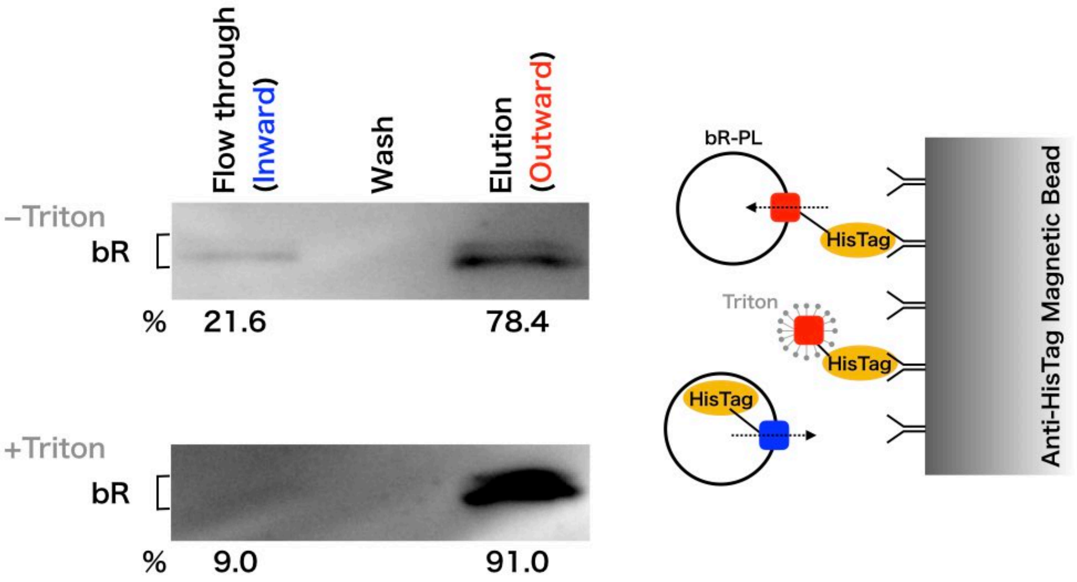

Supplementary Figure 6. Membrane orientation of bacteriorhodopsin (bR) reconstructed in proteoliposome (PL). bR-PLs were prepared with 0.2 μM recombinant bR containing HisTag at the C-terminus and 41.3 mM lipid mixture (SoyPC extract/ cholesterol with the molar ratio 70/30). After mixing with NiNTA-conjugating magnetic beads in the presence or absence of TritonX-100, flow through, wash, and elution fractions were collected and analyzed by SDS-PAGE. The appeared bands in the gel are both bR[2]. The resulting gel was further analyzed by western blotting using anti-HisTag antibody. The bR appeared in the elution fraction represents that C-terminus of bR is facing to the outward of PLs, whereas the bR in the flow through fraction represents that bR is facing to the inward. The light-driven proton flux is indicated by broken arrows. Band intensities were measured by ImageGauge software (Fujifilm).

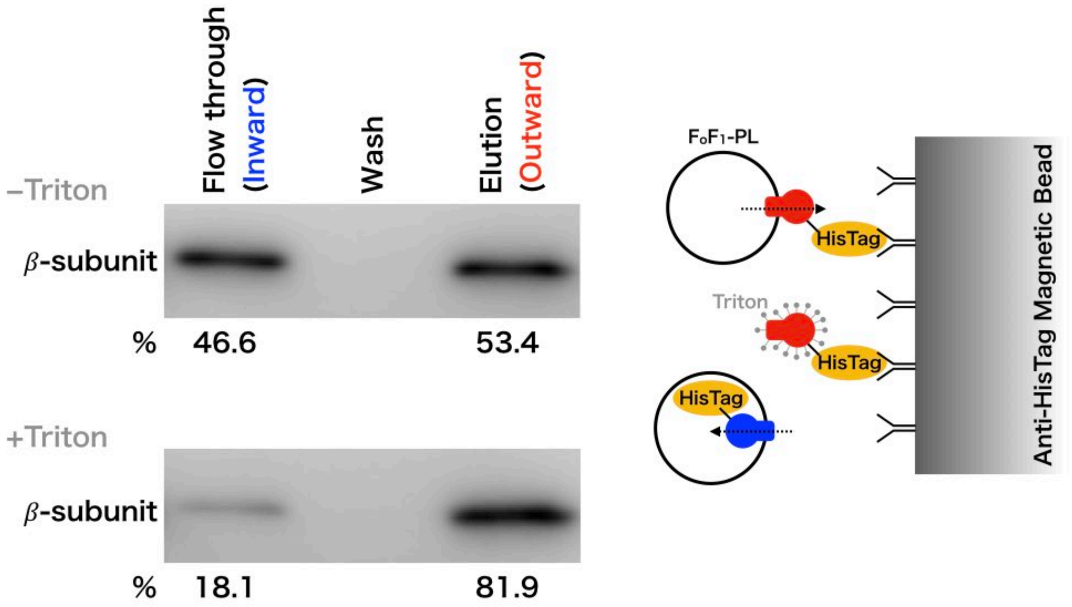

Supplementary Figure 7. Membrane orientation of F_oF_1-ATP synthase (F_oF_1) reconstructed in proteoliposome (PL). F_oF_1-PLs were prepared with 0.2 μM F_oF_1 containing HisTag in β-subunit and 41.3 mM lipid mixture (SoyPC extract/ cholesterol with the molar ratio 70/30). After mixing with NiNTA-conjugating magnetic beads in the presence or absence of TritonX-100, flow through, wash, and elution fractions were separated and analyzed by SDS-PAGE. The resulting gel was further analyzed by western blotting using anti-HisTag antibody to visualize the β-subunit of F_oF_1. The bR appeared in the elution fraction represents that F_1 part is facing to the outward of PLs, whereas the bR in flow through fraction represents that bR is facing to the inward. The proton flux driving ATP synthesis is indicated by broken arrows. Band intensities were measured by ImageGauge software (Fujifilm).

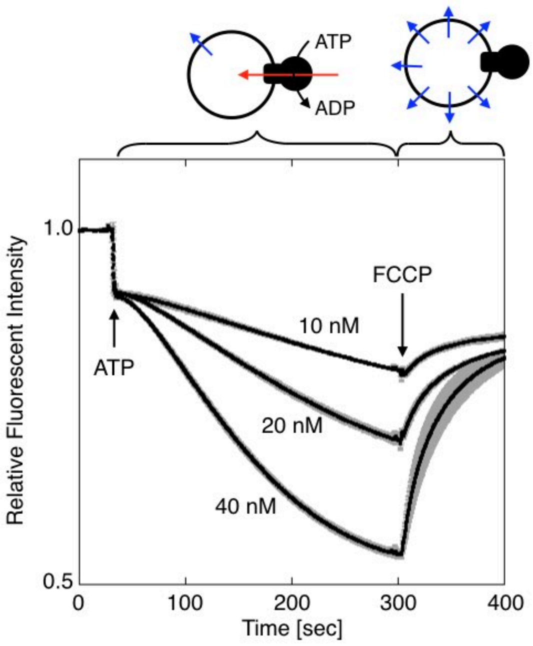

Supplementary Figure 8. ATP-dependent proton-pump activity of F_oF_1. The proton-pump activity of the reconstructed F_oF_1-PLs was measured by monitoring the decrease of proton concentration at the outside of PLs using proton indicator ACMA (ex 410nm/em 480nm). The reaction was triggered by adding ATP at the 30 sec and terminated by adding ionophore FCCP at the 300 sec. The proton flux driven by ATPase activity of F_oF_1 was indicated by red arrow, and proton leak via PLs membrane (left) or FCCP (right) was indicated by blue arrows. The gray shadows indicate STANDARD DEVIATION. from three times measurement.

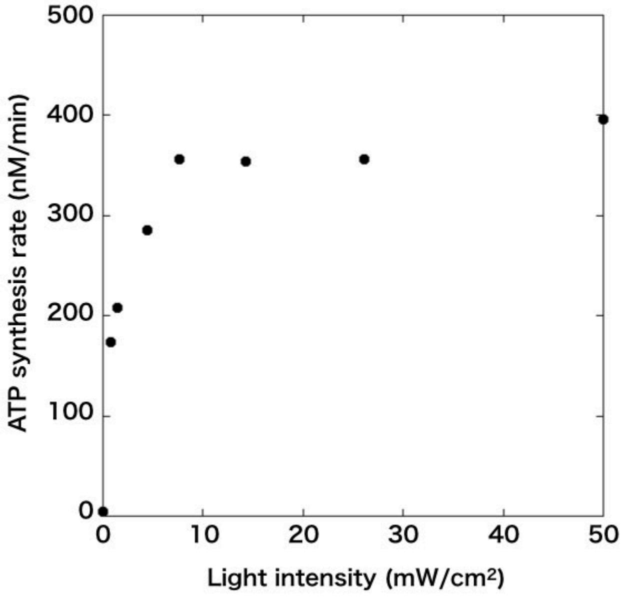

Supplementary Figure 9. Effect of light intensity to the ATP productivity of bRF_oF_1-PLs. ATP synthesis rates (nM/min) were measured using PLs which consists of 146 μM bR and 1 μM F_oF_1. The PLs were illuminated for 10 min at the light intensities in 0, 0.8, 1.4, 4.5, 7.6, 14.3, 26.0, and 50.0 mW/cm^2.

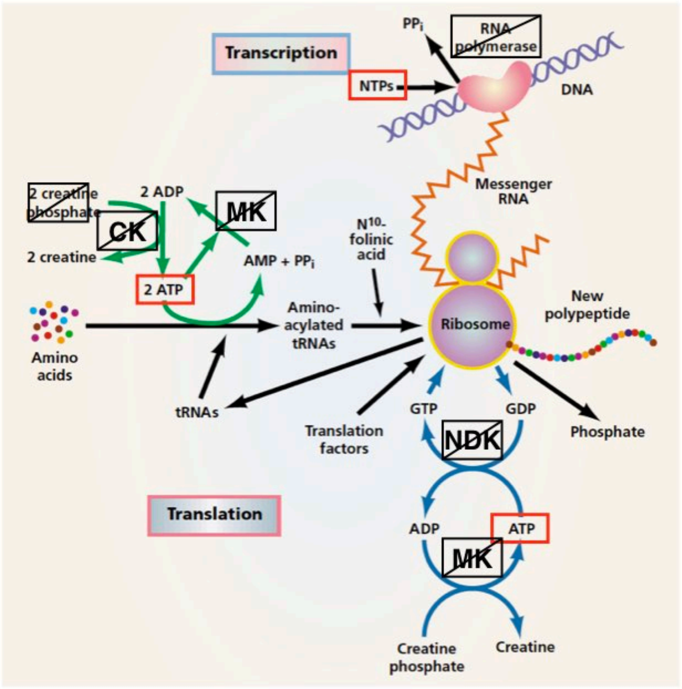

Supplementary Figure 10. ATP consumption in the reconstructed cell-free system (PURE system). The pathways consuming ATP was indicated by red squares; i.e. aminoacylation of tRNAs, phosphorylation of GDP, and transcription. The catalyzing enzymes are described by diagonal squares that were eliminated from the PURE system for light-driven protein synthesis reactions. The figure was modified from[3].

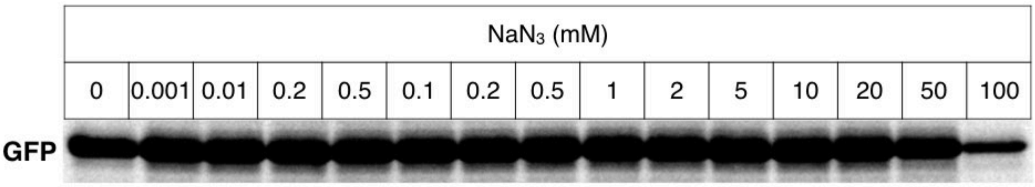

Supplementary Figure 11. Effect of NaN_3 to protein synthesis in the PURE system. GFP was synthesized in the presence of a varied concentrations of NaN_3 and [^{35}S]methionine. The synthesized proteins were analyzed by SDS-PAGE and visualized by autoradiography.

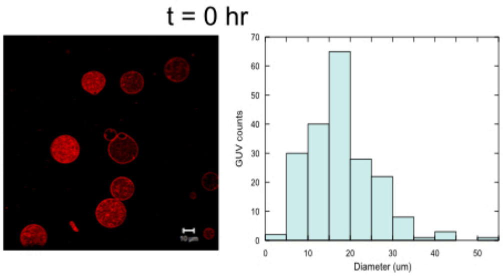

Average	17.9 μm
Median	16.9 μm
S.D.	7.8 μm
N	200

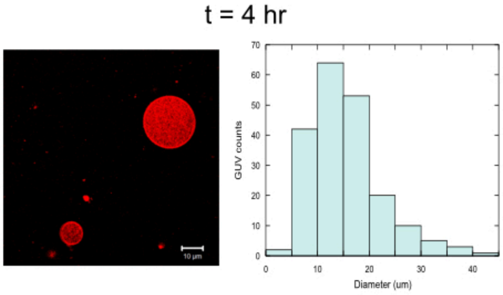

Average	15.4 μm
Median	14.3 μm
S.D.	7.0 μm
N	200

Supplementary Figure 12. Histogram analysis of the size of artificial photosynthetic cell. The size of 200 vesicles containing artificial organelle and PURE system are measured under the microscopy before (t=0) and after 4 hours (t=4) reaction. In both cases, the average size, median, and standard deviation (S.D.) were calculated and shown at the right side of graphs.

bRF_oF_1-PL	+	+	−	+	−	−	−
bR-PL	−	−	−	−	+	−	−
F_oF_1-PL	−	−	−	−	−	+	−
ADP	+	+	+	−	+	+	−
ATP	−	−	−	−	−	−	+
Light	+	−	+	+	+	+	−
Lane	1	2	3	4	5	6	7

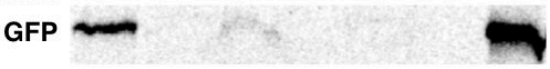

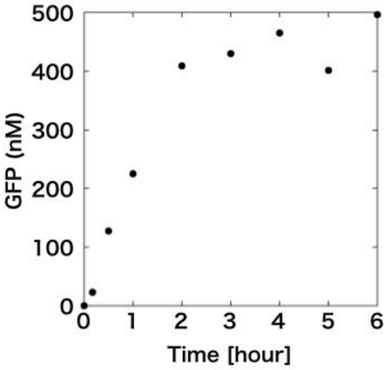

Supplementary Figure 13. Light-driven GFP synthesis in the PURE system containing bRF_oF_1-PLs. The reactions were performed in various conditions at 37 ˚C for 6 hours in the presence of NaN_3, [^{35}S]methionine, and mRNA encoding GFP. The synthesized proteins were analyzed by SDS-PAGE and visualized by autoradiography. Using the lane 1 condition, a time dependent increase of the photosynthesized GFP were analyzed until six hours.

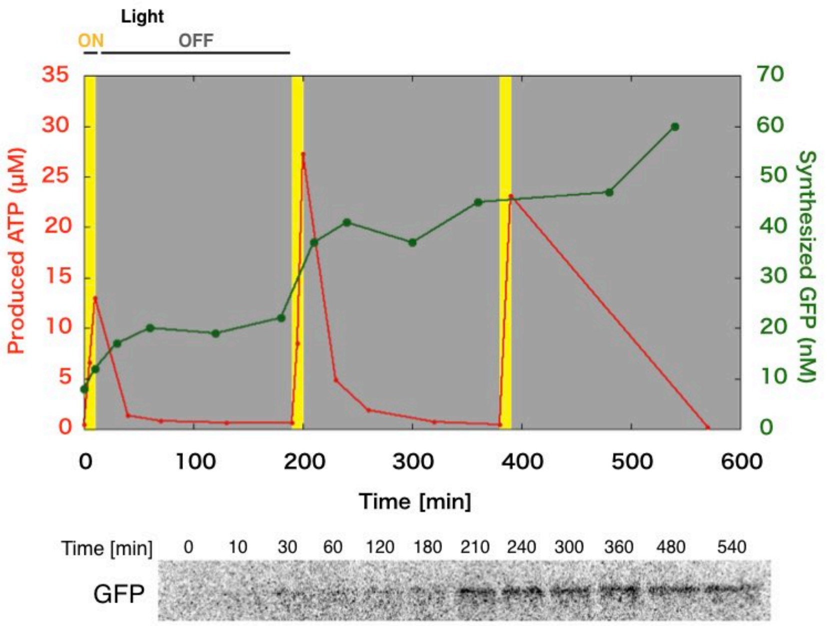

Supplementary Figure 14. Protein synthesis controlled by light irradiation. GFP was photosynthesized in the PURE system containing bRF$_o$F$_1$-PLs and mRNA encoding GFP. The ATP produced by light (yellow zone) was consumed only for aminoacylation of tRNA. The ATP amount was measured by luciferin/luciferase assay (red trace). The amount of synthesized protein was quantified by measuring band intensity of the autoradiography data (green trace).

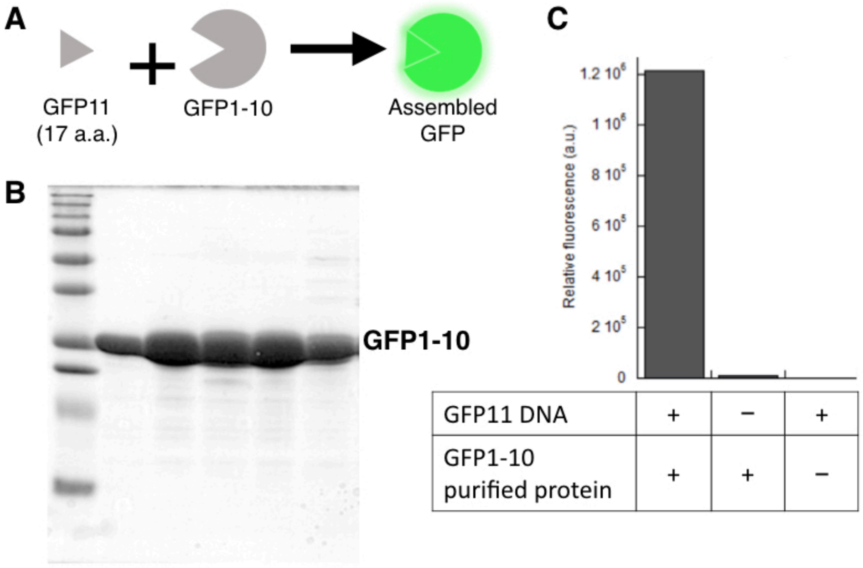

Supplementary Figure 15. Restored fluorescence of the split GFP. (A) Schematics of split GFP. GFP11 consists of 15 amino acid (a.a) and GFP1-10 consist 213 a.a.. The assembled GFP shows fluorescence. **(B)** GFP1-10 purified from *E. coli* cells by NiNTA column followed by MonoQ column purification. **(C)** GFP11 synthesis in PURE system in the presence of the purified GFP1-10 restored a fluorescence as the complete GFP.

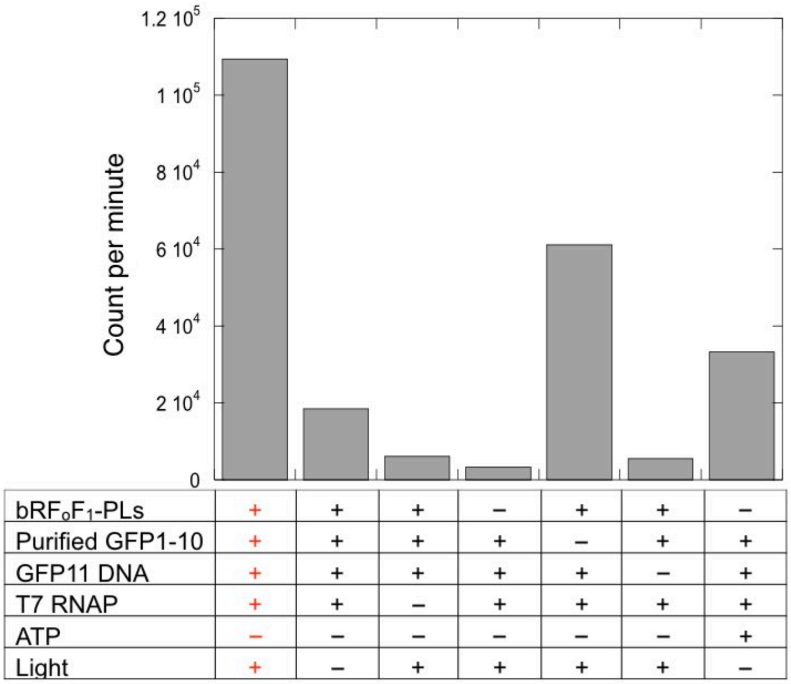

bRF_oF_1-PLs	+	+	+	−	+	+	−
Purified GFP1-10	+	+	+	+	−	+	+
GFP11 DNA	+	+	+	+	+	−	+
T7 RNAP	+	+	−	+	+	+	+
ATP	−	−	−	−	−	−	+
Light	+	−	+	+	+	+	−

Supplementary Figure 16. Light-driven transcription and translation in PURE system containing bRF_oF_1-PLs. GFP11 peptide was synthesized from its DNA in the modified PURE system including bRF_oF_1-PLs, purified GFP1-10, T7 RNAP, NaN_3, and [^{35}S]methionine. The products were separated from free radioactive methionine by filter column. Radioactivity of the resulting samples were counted by scintillation counter.

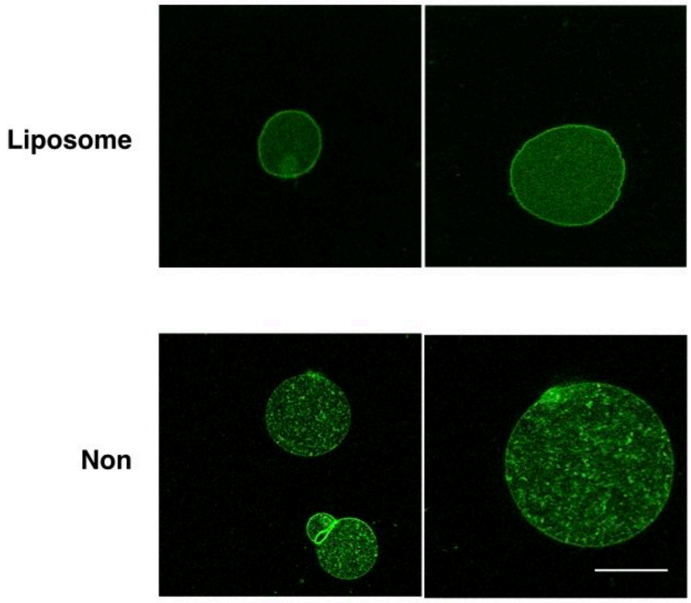

Supplementary Figure 17. bR-GFP synthesis inside GUV containing liposomes. bR, which genetically fused with GFP, was synthesized in the presence or absence of liposomes inside GUVs encapsulating the normal PURE system. Scale bar indicates 20 μm.

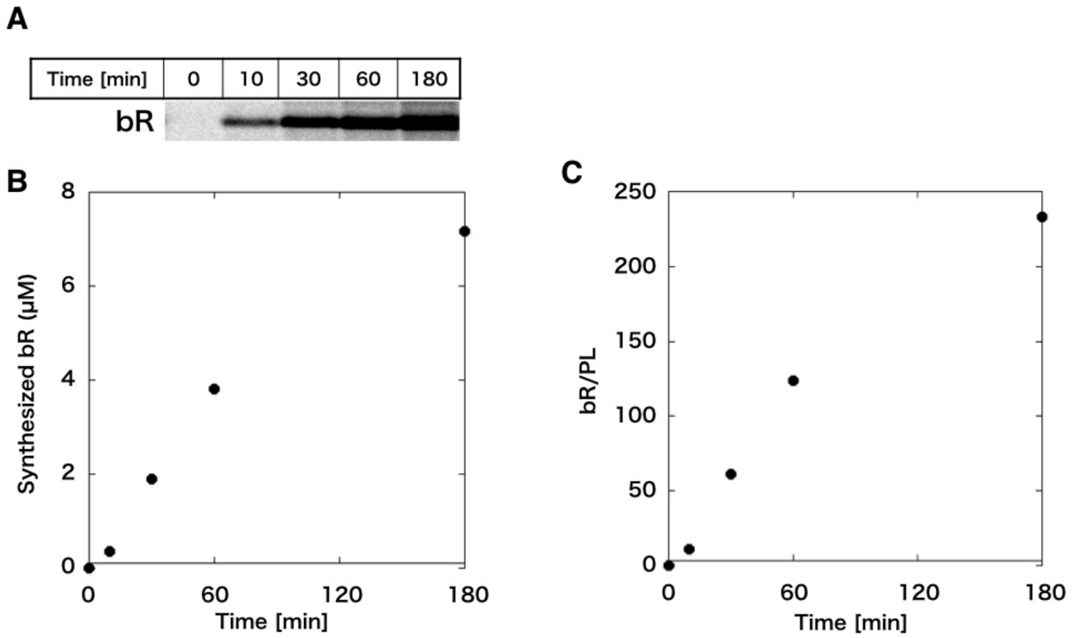

Supplementary Figure 18. Quantification of bR synthesized in the standard PURE system. (A) Wildtype bR was synthesized from its mRNA at the concentration of 400 nM, in the presence of 100 μM retinal. The products were labeled with [^{35}S]methionine. After the reaction, the sample was analyzed by SDS-PAGE and visualized by autoradiography. The band intensities were measured and quantified to obtain bR concentration (μM) **(B)** and the number of bR molecules per liposome **(C)**.

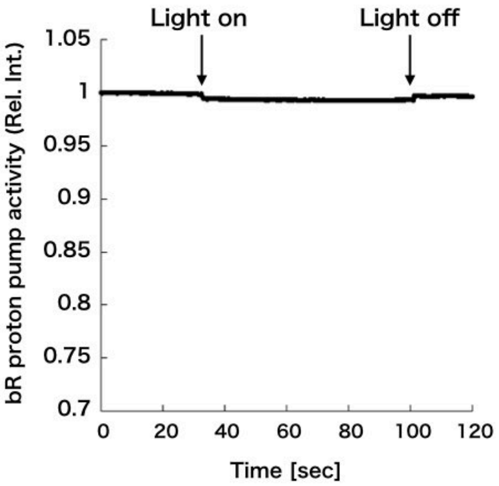

Supplementary Figure 19. Proton-pump activity of the inactive mutant of bR (bR*mut*). bR*mut* was synthesized in the PURE system containing liposomes and retinal. The reacted sample was directly analyzed by a spectrofluorometer by means of proton indicator ACMA (ex 410nm/em 480nm). The proton pump activity was initiated by light on at 30 sec and terminated by light off at 100 sec.

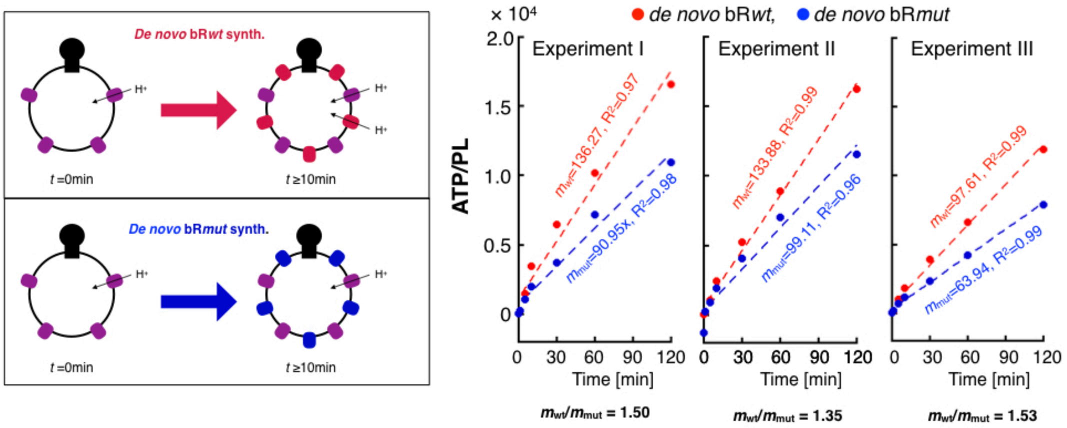

Supplementary Figure 20. Photosynthesis of wildtype or mutant bR in the PURE system containing bRF$_0$F$_1$-PLs. (Left) Schematics of the bRF$_0$F$_1$-PLs reacted in the PURE system which photosynthesizing *de novo* wildtype bR (bR*wt*) or mutant bR (bR*mut*). bRwt or bRmu was photosynthesized from its mRNA. **(Right)** ATP concentration in the PURE system photosynthesizing bR*wt* or bR*mut*. The amounts of ATP were measured at each time point by luciferin/luciferase assay and converted into ATP/PL. Experiments were individually repeated three times.

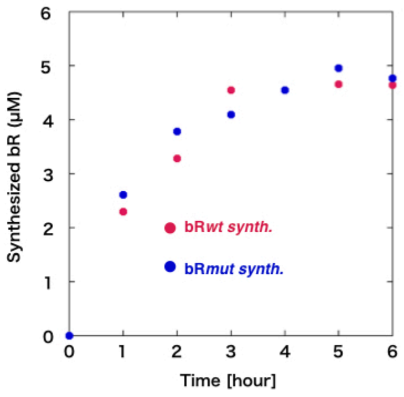

Supplementary Figure 21. Adjusted bR*wt* and bR*mut* synthesis rate. The template mRNA of bR*wt* or bR*mut* was added as 400 or 800 nM, respectively. After the reaction, the synthesized bR were quantified as described in Supplementary Fig. 18 and converted into protein concentration.

	Template DNA conc. (nM)					
a	3	3	3	3	4	4
b	1	1	1	1	2	2
c	3	5	7	10	5	10

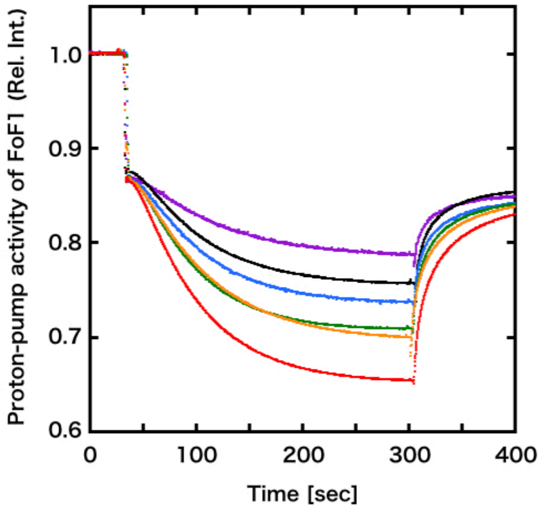

Supplementary Figure 22. ATPase-dependent proton-pump activity of cell-free synthesized F_o in various proportion of template DNA. Various proportions in the DNA concentration of *a*, *b*, and *c*-subunits of F_o were introduced in the PURE system. The products were directly subjected to ATPase-dependent proton-pump assay. The ATP was introduced at 30 sec and the ionophore FCCP was added at 300 sec.

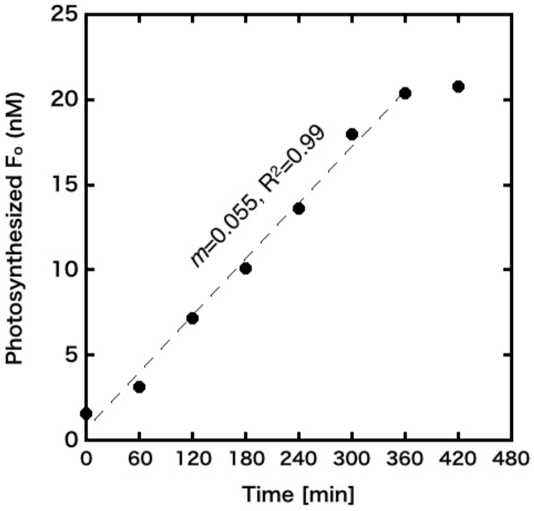

Supplementary Figure 23. F_o photosynthesis in PURE system. Three component proteins of F_o were photosynthesized in the PURE system containing bR$F_o F_1$-PLs. The products were quantified as shown in Supplementary Figure 18 and converted into the photosynthesized F_o concentration.

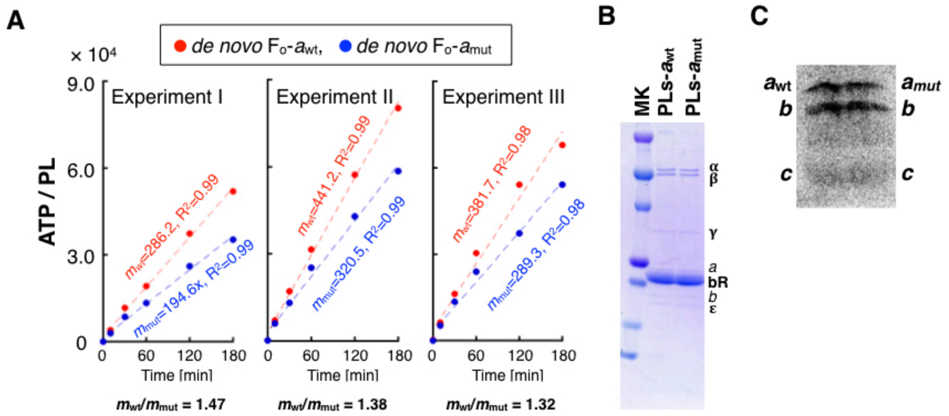

Supplementary Figure 24. Activity enhancement of artificial organelle by positive feedback of the *de novo* photosynthesized F_o. F_o component proteins, a_{wt} (or a_{mut}), *b*, and *c*-subunits were photosynthesized in the PURE system containing bRF_oF_1-PLs. After seven hours reaction, the resulting PLs were isolated from the reaction mixture and illuminated for three hours with supplying ADP. **(A)** ATP amounts in the illuminated mixture were measured by luciferin/luciferase assay and converted into photosynthesized ATP per one PL. The enhancement of photosynthesis activity of PLs was estimated as m_{wt}/m_{mut} and shown below the graphs. Experiment were repeated three times. **(B)** The reacted PLs containing a_{wt} or a_{mut} was equally isolated from the reaction mixture and those component proteins were analyzed by SDS-PAGE. The positions of each proteins are indicated beside of the gel. **(C)** *De novo* photosynthesized a_{wt} (or a_{mut}), *b*, and *c*-subunits labeled with [^{35}S]methionine were analyzed by SDS-PAGE and visualized by autoradiography.

Supplementary Table 1.

Quantitative analysis of components or products in the artificial photosynthetic cell.

Calculation for the number of lipid in a liposome (vesicle)

$$N_{total} = \frac{4\pi r^2 + 4\pi(r-h)^2}{a} \quad (1)$$

Where, N_{total}=average aggregation number of lipid forming a single liposome, r=radius of liposome, h=thickness of lipid bilayer, a=area of the head group of phospholipid.

$$\boldsymbol{N_{lipo}} = \frac{\boldsymbol{M_{lipid} \times NA}}{\boldsymbol{N_{total} \times 1000}} \quad (2)$$

Where, N_{lipo}=number of liposome per ml of solution, M_{lipid}=molar concentration of lipid, NA=6.02 × 10^{23}

Lipid concentration forming liposomes	12 mM (9.4 mg ml^{-1})
Thickness of lipid bilayer (h)	3.8 nm (as DOPC[†])
Head area (a)	0.72 nm^{+2} (as PC[§])
Liposome average radius (r)	100 nm
Number of lipid for one liposome (N_{total})	3.36 × 10^{+5} per Liposome
Total number of liposome (N_{lipo})	2.15 × 10^{+13} mL^{-1}

[†]1,2-dioleoyl-sn-glycerol-3-phosphatidylcholine, [§]phosphatidylcholine

Membrane orientation of the reconstituted component proteins

	Measured ratio (%)	Normalized ratio (%)	Working ratio (%)
F_oF_1-ATP synthase	53.4	65.1	65.1
Bacteriorhodopsin	78.4	86.1	72.2[†]

[†]Reverse proton pumping activity by oppositely oriented bR was reduced from the Normalized ratio.

Specific activity of the component proteins reconstituted in proteoliposomes[*]

F_oF_1-ATP synthase[†]	118±3.2 nmol ATP min^{-1} $nmol^{-1}$	(223±6.12 nmol ATP min^{-1} mg^{-1})
Bacteriorhodopsin[†]	−2.87±0.53 ΔpH min^{-1} $nmol^{-1}$	(−0.11±0.02 ΔpH min^{-1} mg^{-1})

*Considering the ratio of membrane orientation,[†]mean±S.D..

Specific activity of bRF_oF_1-PLs

bR/F_oF_1 (μM)	nmol ATP min^{-1} $nmol^{-1}$ F_oF_1
176/1	522
140/1	355
70/1	276
35/1	226

Population of the component proteins in a single liposome

bR/F_oF_1 (μM)	bR per lipo.	Working bR* (**A**)	F_oF_1 per lipo.	Working F_oF_1* (**B**)	**A/B**
176/1	4930	3560	28	18	198
140/1	3920	2830	28	18	157
70/1	1960	1415	28	18	78
35/1	980	707	28	18	39

*Considering the ratio of membrane orientation.

The working number of bacteriorhodopsin and ATP synthase in a reconstituted proteoliposome

Bacteriorhodopsin (bR)	176 μM
Total number of bR in 1mL	$1.06 \times 10^{+17}$ mL^{-1}
Number of bR per liposome	4930 bR per liposome
Working bR per liposome (mem. ori. correction*)	3560 bR (working) per liposome
F_oF_1-ATP synthase (F_oF_1)	1 μM
Total number of F_oF_1 in 1mL	$6.02 \times 10^{+14}$ mL^{-1}
Number of F_oF_1 per liposome	28 F_oF_1 per liposome
Working F_oF_1 per liposome (mem. ori. correction)	18 F_oF_1 (working) per liposome

*This value was calculated as (176 μM×0.8)–(176 μM×0.2), because oppositely oriented bR pomp out proton to the outside of PL.

ATP synthesis in GUV vs in vitro

	**In GUV**	**In vitro**
Radius (average)	5.0 μm	
Inner volume	0.523 pL	
Amount of the produced ATP	0.95 fmol	
Conc. of the produced ATP	1.8 mM	
Number of PL*	11244	$2.15 \times 10^{+10}$
Produced ATP per PL*	50962±2094	146458±16141

*Artificial organelle consists of bRF_oF_1-PL (176 μM bR per 1 μM F_oF_1)

Supplementary Table 2.

The PURE system enzyme compositions. The enzymes which consume ATP are indicated with red. The eliminated components are indicated with oblique line.

**Standard**			**For mRNA start**			**For DNA start**	
AlaRS	MTF		AlaRS	MTF		AlaRS	MTF
ArgRS	IF1		ArgRS	IF1		ArgRS	IF1
AsnRS	IF2		AsnRS	IF2		AsnRS	IF2
AspRS	IF3		AspRS	IF3		AspRS	IF3
CysRS	EF-G		CysRS	EF-G		CysRS	EF-G
GlnRS	EF-Tu		GlnRS	EF-Tu		GlnRS	EF-Tu
GluRS	EF-Ts		GluRS	EF-Ts		GluRS	EF-Ts
GlyRS	RF1		GlyRS	RF1		GlyRS	RF1
HisRS	RF2		HisRS	RF2		HisRS	RF2
IleRS	RF3		IleRS	RF3		IleRS	RF3
LeuRS	RRF		LeuRS	RRF		LeuRS	RRF
LysRS	MK		LysRS	~~MK~~		LysRS	~~MK~~
MetRS	CK		MetRS	~~CK~~		MetRS	~~CK~~
PheRS	NDK		PheRS	~~NDK~~		PheRS	~~NDK~~
ProRS	PPiase		ProRS	~~PPiase~~		ProRS	~~PPiase~~
SerRS	T7 RNAP		SerRS	~~T7 RNAP~~		SerRS	T7 RNAP
ThrRS	Ribosome		ThrRS	Ribosome		ThrRS	Ribosome
TrpRS			TrpRS			TrpRS	GFP1-10*
TyrRS			TyrRS			TyrRS	
ValRS			ValRS			ValRS	
						*For split-GFP synthesis.	

Supplementary Table 3.

Primer sequences used in the experiments. A broken line and a double line indicate hexa-histidine and T7 promoter sequence, respectively. A bold line indicates ribosome binding site.

Primer	Sequence
P1	GTGATGCAACAATCGGAAAACTTAC
P2	GTAAGTTTTCCGATTGTTGCATCAC
P3	CAAAGATGACGGGAAATACAAGACGCGTG
P4	CACGCGTCTTGTATTTCCCGTCATCTTTG
P5	CAAGACGCGTGCTGTAGTCAAGTTTGAAG
P6	CTTCAAACTTGACTACAGCACGCGTCTTG
P7	GAGTTAAAAGGTACTGATTTTAAAGAAG
P8	CTTCTTTAAAATCAGTACCTTTTAACTC
P9	CAAAGCTAACTTCACAGTTCGCCACAACGTTG
P10	CAACGTTGTGGCGAACTGTGAAGTTAGCTTTG
P11	CTGTCGACACAAACAGTCCTTTCGAAAG
P12	CTTTCGAAAGGACTGTTTGTGTCGACAG
P13	CACATGGTCCTTCATGAGTTTGTAAC
P14	GTTACAAACTCATGAAGGACCATGTG
P15	AAGGAGATATACATATGCATCATCATCATCATCATAGTAAAGGAGAAGAACTTTTCACTG
P16	GACGGAGCTCGAATTCTTACTTTTCGTTGGGATCTTTCG
P17	AAGGAGATATACATATGCGTGACCACATGGTCCTTCATGAGTTTGTAACTGCTGCTGGGATTACATAAGAATTCGAGCTCCGTC
P18	AAGGAGATATACATATGCGTG
P19	GACGGAGCTCGAATTCTT
P20	GGATTAGTTATTCATTATGTAATCCCAGCAGCAGTTAC
P21	GAAATTAATACGACTCACTATAGGGAGACCACAACGGTTTCCCTCTAGAAATAATTTTGTTTAACTTTAAGAAGGAGATATACCA
P22	TAATACGACTCACTATAGGG
P23	GCTAGTTATTGCTCAGCGG
P24	GAAATTAATACGACTCACTATAGGG
P25	ATGCAAGCTCAAATTACTGGACGT
P26	CTTTAAGAAGGAGATATACCAATGCAAGCTCAAATTACTGGACGT
P27	AGTTCTTCTCCTTTACTCATAGAGGTAGCGGCCGCAC
P28	TGCGGCCGCTACCTCTATGAGTAAAGGAGAAGAACTTTTCACT
P29	TTATTTGTAGAGCTCATCCATGCC
P30	GGATTAGTTATTCATTATTTGTAGAGCTCATCCATGCC
P31	GGATTAGTTATTCACTAAGAGGTAGCGGCCGC
P32	GGCCCGTTACGCTAACTGGCTGTTCAC
P33	GTGAACAGCCAGTTAGCGTAACGGGCC
P34	CTAGACGTTTCTGCTAACGTTGGTTTCGGTCTGATC
P35	GATCAGACCGAAAACCAACGTTAGCAGAAACGTCTAG

P36	CTTTAAGAAGGAGATATACCAATGGAGCATAAAGCGCCG
P37	GGATTAGTTATTCATTAATGGTCATGGCTGACCTTATG
P38	CTTTAAGAAGGAGATATACCAATGTTGTGGAAGGCAAACGTATG
P39	GGATTAGTTATTCATTAGCTTGCCGCTTGATCTTGCTC
P40	CTTTAAGAAGGAGATATACCAATGAGTTTGGGTGTACTTGC
P41	GGATTAGTTATTCATTATCGACCTAAGTAAATGAACGAG
P42	TGACGCTCGGTTTGGCTCTTTTCGGGAACATTTACGCC
P43	GCGTAAATGTTCCCGAAAAGAGCCAAACCGAGCGTCAGCGT

Supplementary Table 4.

The PURE system buffer compositions.

Component	Concentration		
	**Buffer-1[*] [pH 7.3]**	**Buffer-2[†] [pH 7.3]**	**Buffer-3[§] [pH 7.3]**
HEPES-KOH (pH 7.6)	10 mM	10 mM	10 mM
K-Glutamate	180 mM	180 mM	180 mM
$Mg(OAc)_2$	18 mM	18 mM	18 mM
NaH_2PO_4	10 mM	10 mM	10 mM
Creatine Phosphate	60 mM	60 mM	60 mM
10-Formyl-5,6,7,8-tetrahydrofolic acid	10 µg/ ml	10 µg/ ml	10 µg/ ml
Spermidine	2 mM	2 mM	2 mM
Dithiothreitol	2 mM	2 mM	2 mM
18 amino acids mix (w/o Cys&Tyr)	2 mM	2 mM	2 mM
Cysteine	1 mM	1 mM	1 mM
Tyrosine	1 mM	1 mM	1 mM
GTP	3 mM	3 mM	0 mM
UTP	0 mM	1 mM	0 mM

*For light-driven translation system; †For light-driven transcription-and-translation coupled system; §For light-driven GTP synthesis for the translation system. The pH of each buffers was adjusted with HCl.

Supplementary Table 5.

List of DNA sequences cloned and used in this research. Single and double under lines indicate T7 promoter and ribosome binding site, respectively. Open reading frames are shown by bold texts.

1. pET21c-bR_wt<br>TAATACGACTCACTATAGGGGAATTGTGAGCGGATAACAATTCCCCTCTAG<br>AAATAATTTTGTTTAACTTTAAGAAGGAGATATACAT**ATGTTGGAGTTATTG**<br>**CCAACAGCAGTGGAGGGGGTATCGCAGGCCCAGATCACCGGACGTCC**<br>**GGAGTGGATCTGGCTAGCGCTCGGTACGGCGCTAATGGGACTCGGGAC**<br>**GCTCTATTTCCTCGTGAAAGGGATGGGCGTCTCGGACCCAGATGCAAA**<br>**GAAATTCTACGCCATCACGACGCTCGTCCCAGCCATCGCGTTCACGAT**<br>**GTACCTCTCGATGCTGCTGGGGTATGGCCTCACAATGGTACCGTTCGG**<br>**TGGGGAGCAGAACCCCATCTACTGGGCGCGGTACGCTGACTGGCTGT**<br>**TCACCACGCCGCTGTTGTTGTTAGACCTCGCGTTGCTCGTTGACGCGG**<br>**ATCAGGGAACGATCCTTGCGCTCGTCGGTGCCGACGGCATCATGATCG**<br>**GGACCGGCCTGGTCGGCGCACTGACGAAGGTCTACTCGTACCGCTTC**<br>**GTGTGGTGGGCGATCAGCACCGCAGCGATGCTGTACATCCTGTACGTG**<br>**CTGTTCTTCGGGTTCACCTCGAAGGCCGAAAGCATGCGCCCCGAGGTC**<br>**GCATCCACGTTCAAAGTACTGCGTAACGTTACCGTTGTGTTGTGGTCCG**<br>**CGTATCCCGTCGTGTGGCTGATCGGCAGCGAAGGTGCGGGAATCGTG**<br>**CCGCTGAACATCGAGACGCTGCTGTTCATGGTGCTTGACGTGAGCGCG**<br>**AAGGTCGGCTTCGGGCTCATCCTCCTGCGCAGTCGTGCGATCTTCGGC**<br>**GAAGCCGAAGCGCCGGAGCCGTCCGCCGGCGACGGCGCGGCCGCGA**<br>**CCAGCGACCACCACCACCACCACCACTGA**
2. pEXP5-CT/bR_D85N/K216N<br>TAATACGACTCACTATAGGGGAATTGTGAGCGGATAACAATTCCCCTCTAG<br>AAATAATTTTGTTTAACTTTAAGAAGGAGATATACAT**ATGCAAGCTCAAATT**<br>**ACTGGACGTCCGGAATGGATCTGGCTAGCTCTGGGCACCGCTCTGATG**<br>**GGTCTGGGCACCCTGTACTTCCTGGTTAAAGGTATGGGTGTTTCGGATC**<br>**CGGATGCGAAAAAATTCTACGCTATCACCACCCTGGTGCCGGCTATCGC**<br>**ATTCACCATGTACCTGTCTATGCTGCTGGGTTACGGTCTGACCATGGTAC**<br>**CGTTCGGTGGTGAACAGAACCCGATCTACTGGGCCCGTTACGCTAACT**

GGCTGTTCACCACCCCGCTGCTGCTAGATCTGGCTCTGCTGGTTGA
CGCTGATCAGGGCACCATCCTGGCTCTGGTTGGCGCCGACGGTATCAT
GATCGGCACCGGCCTGGTTGGCGCGCTGACCAAGGTTTACTCTTACCG
TTTCGTTTGGTGGGCTATCTCTACTGCAGCTATGCTGTACATCCTGTACG
TACTGTTCTTCGGTTTCACCTCTAAAGCTGAAAGCATGCGTCCGGAAGT
TGCGTCGACCTTCAAAGTACTGCGTAACGTTACCGTTGTTCTGTGGTCC
GCTTACCCAGTTGTTTGGCTGATCGGTTCTGAAGGTGCCGGCATTGTTC
CGCTGAATATTGAAACCCTGCTGTTCATGGTTCTAGACGTTTCTGCTAAC
GTTGGTTTCGGTCTGATCCTGCTGCGTTCTCGAGCTATCTTCGGTGAAG
CTGAAGCTCCGGAACCGTCCGCGGGTGACGGTGCGGCCGCTACCTCT
TAG

3. pET29a-bR::sfGFP

TAATACGACTCACTATAGGGGAATTGTGAGCGGATAACAATTCCCCTCTAG
AAATAATTTTGTTTAACTTTAAGAAGGAGATATACAT**ATGCAAGCTCAAATT**
ACTGGACGTCCGGAATGGATCTGGCTAGCTCTGGGCACCGCTCTGATG
GGTCTGGGCACCCTGTACTTCCTGGTTAAAGGTATGGGTGTTTCGGATC
CGGATGCGAAAAAATTCTACGCTATCACCACCCTGGTGCCGGCTATCGC
ATTCACCATGTACCTGTCTATGCTGCTGGGTTACGGTCTGACCATGGTAC
CGTTCGGTGGTGAACAGAACCCGATCTACTGGGCCCGTTACGCTGACT
GGCTGTTCACCACCCCGCTGCTGCTAGATCTGGCTCTGCTGGTTGA
CGCTGATCAGGGCACCATCCTGGCTCTGGTTGGCGCCGACGGTATCAT
GATCGGCACCGGCCTGGTTGGCGCGCTGACCAAGGTTTACTCTTACCG
TTTCGTTTGGTGGGCTATCTCTACTGCAGCTATGCTGTACATCCTGTACG
TACTGTTCTTCGGTTTCACCTCTAAAGCTGAAAGCATGCGTCCGGAAGT
TGCGTCGACCTTCAAAGTACTGCGTAACGTTACCGTTGTTCTGTGGTCC
GCTTACCCAGTTGTTTGGCTGATCGGTTCTGAAGGTGCCGGCATTGTTC
CGCTGAATATTGAAACCCTGCTGTTCATGGTTCTAGACGTTTCTGCTAAA
GTTGGTTTCGGTCTGATCCTGCTGCGTTCTCGAGCTATCTTCGGTGAAG
CTGAAGCTCCGGAACCGTCCGCGGGTGACGGTGCGGCCGCTACCTCT
ATGAGTAAAGGAGAAGAACTTTTCACTGGAGTTGTCCCAATTCTTGTTG
AATTAGATGGTGATGTTAATGGGCACAAATTTTCTGTCCGTGGAGAGGG
TGAAGGTGATGCAACAAACGGAAAACTTACCCTTAAATTTATTTGCACT

ACTGGAAAACTACCTGTTCCATGGCCAACACTTGTCACTACTTTAACTTA
TGGTGTTCAATGCTTTTCCCGTTATCCGGATCACATGAAACGGCATGAC
TTTTTCAAGAGTGCCATGCCCGAAGGTTATGTACAGGAACGCACTATAT
CTTTCAAAGATGACGGGACCTACAAGACGCGTGCTGAAGTCAAGTTTG
AAGGTGATACCCTTGTTAATCGTATCGAGTTAAAAGGTATTGATTTTAAA
GAAGATGGAAACATTCTCGGACACAAACTCGAGTACAACTTTAACTCAC
ACAATGTATACATCACGGCAGACAAACAAAAGAATGGAATCAAAGCTAA
CTTCAAAATTCGCCACAACGTTGAAGATGGATCCGTTCAACTAGCAGAC
CATTATCAACAAAATACTCCAATTGGCGATGGCCCTGTCCTTTTACCAGA
CAACCATTACCTGTCGACACAATCTGTCCTTTCGAAAGATCCCAACGAA
AAGCGTGACCACATGGTCCTTCTTGAGTTTGTAACTGCTGCTGGGATTA
CACATGGCATGGATGAGCTCTACAAATAA

4. pET29a-GFP1-10

TAATACGACTCACTATAGGGGAATTGTGAGCGGATAACAATTCCCCTCTAG
AAATAATTTTGTTTAACTTTAAGAAGGAGATATACAT**ATGCATCATCATCAT**
CATCATAGTAAAGGAGAAGAACTTTTCACTGGAGTTGTCCCAATTCTTG
TTGAATTAGATGGTGATGTTAATGGGCACAAATTTTCTGTCCGTGGAGA
GGGTGAAGGTGATGCAACAATCGGAAAACTTACCCTTAAATTTATTTGC
ACTACTGGAAAACTACCTGTTCCATGGCCAACACTTGTCACTACTTTAA
CTTATGGTGTTCAATGCTTTTCCCGTTATCCGGATCACATGAAACGGCAT
GACTTTTTCAAGAGTGCCATGCCCGAAGGTTATGTACAGGAACGCACTA
TATCTTTCAAAGATGACGGGAAATACAAGACGCGTGCTGTAGTCAAGTT
TGAAGGTGATACCCTTGTTAATCGTATCGAGTTAAAAGGTACTGATTTTA
AAGAAGATGGAAACATTCTCGGACACAAACTCGAGTACAACTTTAACTC
ACACAATGTATACATCACGGCAGACAAACAAAAGAATGGAATCAAAGC
TAACTTCACAGTTCGCCACAACGTTGAAGATGGATCCGTTCAACTAGCA
GACCATTATCAACAAAATACTCCAATTGGCGATGGCCCTGTCCTTTTACC
AGACAACCATTACCTGTCGACACAAACAGTCCTTTCGAAAGATCCCAA
CGAAAAGTAA

5. pET29a-GFP11_L221H

TAATACGACTCACTATAGGGGAATTGTGAGCGGATAACAATTCCCCTCTAG
AAATAATTTTGTTTAACTTTAAGAAGGAGATATACAT**ATGCGTGACCACATG**

**GTCCTTCATGAGTTTGTAACTGCTGCTGGGATTACATAA**
6. pET32a-sfGFP<br>TAATACGACTCACTATAGGGGAATTGTGAGCGGATAACAATTCCCCTCTAG<br>AAATAATTTTGTTTAACTTTAAGAAGGAGATATACAT**ATGAGTAAAGGAGA**<br>**AGAACTTTTCACTGGAGTTGTCCCAATTCTTGTTGAATTAGATGGTGATG**<br>**TTAATGGGCACAAATTTTCTGTCCGTGGAGAGGGTGAAGGTGATGCAA**<br>**CAAACGGAAAACTTACCCTTAAATTTATTTGCACTACTGGAAAACTACCT**<br>**GTTCCATGGCCAACACTTGTCACTACTTTAACTTATGGTGTTCAATGCTT**<br>**TTCCCGTTATCCGGATCACATGAAACGGCATGACTTTTTCAAGAGTGCC**<br>**ATGCCCGAAGGTTATGTACAGGAACGCACTATATCTTTCAAAGATGACG**<br>**GGACCTACAAGACGCGTGCTGAAGTCAAGTTTGAAGGTGATACCCTTG**<br>**TTAATCGTATCGAGTTAAAAGGTATTGATTTTAAAGAAGATGGAAACATT**<br>**CTCGGACACAAACTCGAGTACAACTTTAACTCACACAATGTATACATCA**<br>**CGGCAGACAAACAAAAGAATGGAATCAAAGCTAACTTCAAAATTCGCC**<br>**ACAACGTTGAAGATGGATCCGTTCAACTAGCAGACCATTATCAACAAAA**<br>**TACTCCAATTGGCGATGGCCCTGTCCTTTTACCAGACAACCATTACCTGT**<br>**CGACACAATCTGTCCTTTCGAAAGATCCCAACGAAAAGCGTGACCACA**<br>**TGGTCCTTCTTGAGTTTGTAACTGCTGCTGGGATTACACATGGCATGGAT**<br>**GAGCTCTACAAATAA**

Supplemental references

1 Pitard, B., Richard, P., Dunach, M., Girault, G. & Rigaud, J. L. ATP synthesis by the F0F1 ATP synthase from thermophilic Bacillus PS3 reconstituted into liposomes with bacteriorhodopsin. 1. Factors defining the optimal reconstitution of ATP synthases with bacteriorhodopsin. *European journal of biochemistry* **235**, 769-778 (1996).

2 Miercke, L. J., Ross, P. E., Stroud, R. M. & Dratz, E. A. Purification of bacteriorhodopsin and characterization of mature and partially processed forms. *The Journal of biological chemistry* **264**, 7531-7535 (1989).

3 Swartz, J. A PURE approach to constructive biology. *Nature biotechnology* **19**, 732-733, (2001).
